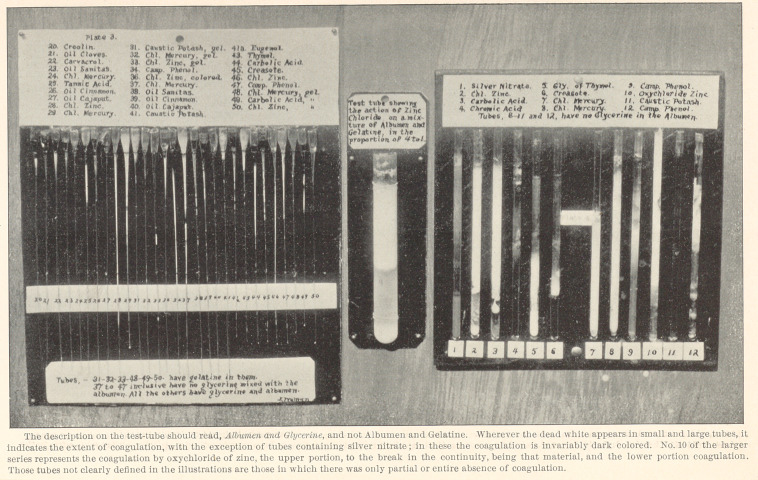# The Relative Penetrating Power of Coagulants

**Published:** 1895-01

**Authors:** James Truman

**Affiliations:** Philadelphia


					﻿
THE RELATIVE PENETRATING POWER OF COAGU-
LANTS.¹

¹ Read before the Academy of Stomatology, Philadelphia, December 10,1894.

BY JAMES TRUMAN, PHILADELPHIA.

    The question of the proper material for filling root-canals has
been a subject of discussion and experiment for the past half-cen-
tury, or since Maynard introduced the method of filling these canals
with gold. In connection with this the consideration of certain
agents has led to a decided antagonism in regard to the diffusibility,
coagulation, and antiseptic properties and values to be placed upon
these in the general treatment of the central pulp-canal and the
tubuli branching from this throughout the dentine.
    It has been clearly evident that the inner tubular portion, fibres
of Tomes and Neumann sheaths, form no insignificant part of the
organic matter of the tooth substance, and that death of the cen-
tral organ means necessarily the death of the whole and subse-
quent decomposition of this tissue, or at least the central proto-
plasmic portion, the sheaths being almost indestructible. Hence
the treatment of the pulp-canal, however perfectly accomplished,
must fail to reach the microscopic elements in the tubes, and the
decomposition taking place therein results in the discoloration of
the entire tooth, and may act disastrously by septic emanations
upon the vitality of the entire structure. The importance of this
has not been lost sight of by intelligent operators, but the difficul-
ties of manipulation have been serious. It has been plain that but
two methods could be relied upon to overcome the difficulty, the
property of coagulation and the diffusibility of various essential
oils, aided by osmotic action. Both methods have had decided ad-
vocacy and it is very probable that both have a positive value, the
extent of which has as yet to be determined, for, as far as I am
aware, the relative values of the systems of treatment have not
been settled with satisfactory experimentation, or, if so, have not
been divulged in the papers upon this subject; all the points defended




by the writers seem almost entirely to be based on assumptions,
imperfect experiments, or upon clinical observations.
    The difficulties surrounding the subject, and the many errors of
observation to which experimentation in this direction is liable, has
led to criticism, and have thrown a shadow of doubt upon those
made by several observers.
    It is not the purpose of tlris article to enter into any contention
with the two schools of thought on this subject, but rather to show,
if it be possible, that the arguments maintained with so much vigor
and pertinacity that coagulation furnishes its own barrier to diffu-
sion is an error of observation. Further, it is desired to demon-
strate that the various coagulants have relative degrees of value.
This has seemed to possess some degree of importance in other
directions than that of coagulation of albuminoid material in
dentine.
    The knowledge on this subject seems, as far as I am aware,
somewhat crude. The general idea being to rest satisfied with the
fact whether a certain agent will coagulate or not, and in many
instances writers have ascribed this property to agents that do not
possess it. There seems, therefore, good reason for an attempt to
settle several questions by careful experiment.
    For a clear understanding of this subject a brief resume of the
opinions of various writers may not be out of place as a preface to
the present paper.
    Dr. G-. V. Black, who has rendered valuable service in bis report
on the value of essential oils, makes this assertion in the discussion
following a paper by Dr. A. W. Harlan on “The Action of Diffusi-
ble Medicinal Agents on Living Teeth” (Dental Review, June, 1891).
“ If it is a microbe that attacks the sarcous elements of the body,
the contents of the dental tubules are attacked just as well. So
here we need a disinfectant. What shall we use? Certainly not
one of the coagulants, certainly not one that places a barrier to its own
penetration by coagulating the albumen, as does carbolic acid, bichloride
of mercury, and some others. These are not the antiseptics you should
use in this place, but something that is diffusible, that docs not co-
agulate albumen, something we can depend upon to penetrate in
the presence of albumen, and we find that to-day in the use of the
essential oils.”
    In the report on Materia Medica and Therapeutics, by Dr. Harlan
(American Dental Association, 1889), he makes the following state-
ment: “It is not denied here that coagulants are useful medica-
ments in dental practice, but we maintain that in the treatment of

pulpless teeth for purposes of disinfection, they are not only useless
but detrimental, in fact, their use defeats the object sought to be
attained. They coagulate the organic surface with which they come in
contact and prevent their own diffusion.”
    Again, in the paper before alluded to (Dental Review, June,
1891), he states that, “ The coagulating agents simply coagulate
and leave the coagulum to become a source of food-supply to the
aerobic and anaerobic microbes. ... In all my experiments on teeth
out of the mouth, the coagulators, in not a single instance, passed
through tbe dentine; as soon as the concretion of the surface of the
cavity was affected their action was stopped.” After describing
the method to prove diffusibility, he says, “In this manner I have
found that all volatile oils and turpentine will pass through dentine
and cementum, but carbolic acid, creosote, chloride of zinc, and
aromatic sulphuric acid will not pass through the dentine or ce-
mentum.”
    Dr. Hugenschmidt, in his paper on “ The Conservative Treat-
ment of the Dental Pulp,” endorses this idea, for he states that,
“ When we apply a coagulating antiseptic, we, of course, set up a
barrier to the further action of the remedy, as has been repeatedly
and correctly stated by our confrere, Dr. Harlan.”
    For the present it is proposed to leave these various opinions as
they stand, though I shall have occasion to refer to them in tbe
course of the paper.
    In a discussion on root-filling before the American Dental Asso-
ciation, held at Saratoga Springs, 1889 (Transactions'), I made the
following remarks : “ The question that has given me much thought
for the past two or three years is that no filling of canals can be per-
fect. Why ? Because a large part of the tooth is made up of tubu-
lated structure, the tubuli holding organic matter, and when the
pulp dies the organic substance dies and decomposition begins im-
mediately, and the sulphuretted hydrogen occasioned by that decom-
position is the principal source of trouble. What should be done?
My study of the subject and the results of many experiments led
me to the conclusion that it was essential to prevent this decompo-
sition, and that this could be best accomplished by coagulating the
organic tissue. The coagulant best adapted for this purpose is
chloride of zinc, because of its great affinity for water. It will
follow moisture to the very extremities of the tubulated structure,
and consequently change the character of the contents so that de-
composition will not take place. I have kept coagulated albumen
for months without change.”

    This quotation comprises the substance of my views held five
years ago. On the principal points the same ideas are held
to-day.
    Dr. Edward C. Kirk, in a paper “ On Coagulants in the Treat-
ment of the Pulp-Chamber and Canals” (Dental Cosmos, March,
1894), takes the positive ground in favor of the use of coagulants,
using the following language: “ The use of coagulants, such as
carbolic acid, zinc chloride, sublimate, etc., has not only over-
whelming clinical evidence in its favor . . . but it is actually proper
on theoretical grounds, and fully in harmony with the laws of osmo-
sis.” His experiments wrere so carefully made that they seemed, to
candid minds, to have settled the question, and yet they have not
proved convincing to the opposite side of the question, for reasons
not as yet clearly defined or at least not understood.
    The direction of this present paper is only incidentally aimed
as answer to the assertion that coagulants act as a bar to their own
diffusion, for it has to do principally with the proposition, To what
extent can this coagulation be relied upon to effect satisfactory
results ?
    The position taken by myself in 1889, that coagulants placed in
the central canal would permeate the tubuli and coagulate the
contents, remains true to-day, as the experiments in capillary
tubes will demonstrate, and as was previously also demonstrated
by Dr. Kirk, reported in the Dental Cosmos. It is, therefore, use-
less to combat the ideas entertained in the quotations of an op-
posing character, as they have no force. The question might be
left where it was placed by those experiments, but it seemed to me
there was something more to be said on this question not entered
into by Dr. Kirk. Some of these points were taken up seven years
ago by myself, but dropped for a more favorable opportunity to
continue them.
    My intention was to endeavor to show that coagulants would
penetrate tubes of minutest character possible to be bandied satis-
factorily, and that this penetration was independent of circulation.
My earlier investigations seemed to warrant this belief. Diffusion
is recognized in the living tooth as performing an important and
continuous part in its nutrition. It seemed certain, as the tubulated
portion of the dentine invariably imbibed finely-divided colored
matter in solution, that therefore it must take up any other fluid,
if of equal solubility, with the same facility. This beyond question
is true. The main difficulty here being to demonstrate that the
coagulation was continuous without the aid of circulation.

    The early experiments abundantly proved this to be true, but
they were carried on, at the time, with difficulty.
    The effort was, as before stated, to find results in tubes not ex-
ceeding a millimetre in diameter, and if coagulation occurred it
must be through absolute contact of the agent with the albumen or
gelatin used in the experiment. It was necessary to fill the minute
tubes with the albumen and then seal the ends. Both processes
were accomplished readily by nearly filling the tubes and then
quickly melting the ends in a Bunsen burner. This proved entirely
satisfactory. It was found, however, that the albumen in the tubes
dried and contracted upon itself, leaving spaces. To meet this
difficulty the albumen was combined with twenty per cent, of glyc-
erin. This served an excellent purpose, and proved no interference
with coagulation either in large or small tubes, with all the agents
known to be positive coagulants, with one exception. It was found
that mercuric chloride had little or no effect apparently on albumen
and glycerin. This was repeated a number of times. It was then
applied to albumen without glycerin, and coagulation was imme-
diate. It was found, however, that glycerin simply delayed coagu-
lation, for in the course of a few days the effect of the mercuric
chloride was plainly visible in flocculent masses.
    This fact necessitated a repetition of all the experiments to de-
termine their correctness. It was found that mercuric chloride
was the principal one of the series seriously antagonized by the
glycerin.
    These experiments have occupied several months, as the con-
clusions were not arrived at until constant repetitions, under vary-
ing conditions, had demonstated their correctness.
    The tubes were drawn to varying lengths not exceeding, as a
rule, over 0.5 millimetre in diameter. The unit of time was fixed
at ten days. The first series exhibited some variation in the num-
ber of centimetres, but as the measurements of the fluids had not
been exact, it was determined to try the most important coagulants
a number of times with greater accuracy. This gave more satis-
factory results, showing in Plate No. 2 very little variation in
duplicate, while in Plate No. 1, there is a difference of fully a
centimetre in some of the tubes.
    The experiments were also made to include the essential oils
and many agents known to be non-coagulants, for the reason that
some writers have asserted that several of these produced coagulum.
    Further, the action on gelatin was examined into, but this was
confined to but few tubes and without marked result.

        A portion of this work is given in the following table, repre-
    senting as it does some of the most important coagulants in use:

        When the results of these experiments are analyzed, it is found,
     taking Plate 2 as the best representation, that no results were
     attainable with chromic acid. This was tried repeatedly in the
     small tubes, 5, Plate 1, and 11, Plate 2, also in 4, Plate 3 (large
     tubes). Silver nitrate exhibited thorough coagulation, 7, 12, 16,
     Plates 1 and 2, and tube 1, Plate 3. With zinc chloride the coagu-
     lation is complete in 2, 3, 13, 17, Plates 1 and 2, and 28 and 46,
     Plate 3, and tube 2, Plate 4, and also in tbe large test-tube. Car-
     bolic acid shows partial coagulation in all the small tubes, but com-
     plete in No. 3, Plate 4. This last tube, however, was not started
     properly, owing to difference in density of the two liquids causing

them to mix to some extent. The variation in centimetres in ten
days, Plate 2, is very slight, the lengths being in No. 14, 2.8 cen-
timetres, and in 18, 2.6 centimetres.
    Whenever possible the effort was made to have coagulation pro-
ceed in opposition to gravity. This is beautifully shown in the test-
tube.
    What does the work as a whole teach ?   1. That coagulants do
not prevent by their own action the diffusion throughout the entire
tube.
    2.    That the penetrating power of such agents as creosote, car-
bolic acid, and zinc chloride, those most frequently used, varies
materially. That creosote is a very poor coagulant when compared
with carbolic acid, and the latter, for this purpose, is not to be com-
pared with zinc chloride or silver nitrate.
    3.    That in proportion to the coagulating power of the agent
will be its penetrating force independent of gravitation.
    No attempt was made to determine with exactness the pene-
trating property of essential oils, but if coloration is any indica-
tion, the tubes presented do not indicate that this is of much value
in closed tubes, but this, it is acknowledged, may not apply in tubes
where circulation is an adjunct to aid penetration.
    In order to test various agents, such as the essential oils, and
also to repeat the tests with agents already used, as carbolic acid,
chloride of zinc, creosote, etc., with albumen and glycerin, and
without glycerin, and also the effect on gelatin, the series of tubes
ranging from 20 to 50 were prepared.
   It will be observed that creolin gives but slight coagulating
   Q                                             O       O      O
effect, oil of cloves about the same, carvacrol shows slight cloudi-
ness, sanitas oil slight coagulation, mercuric chloride no coagulation
in this tube (glycerin and albumen). Tannic acid shows extended
coagulation; oil of cinnamon, action marked but limited; oil of
cajaput, no result; caustic potash, no result in this or other tubes ;
zinc chloride in gelatin, no result; phenol sodique, partial coagula-
tion. On No. 36 an attempt was made to carry staining with the
coagulation. Zinc chloride was colored with carmine, with the re-
sult that the coagulation left the stain and proceeded down the
tube. Eugenol is but a poor coagulator, shown in 41g. On thymol,
the effect is but slight and not continuous.
    Tube 10, Plate 4, is given to show the possible action of oxy-
chloride of zinc on the contents of the tubuli in the dentine. The
oxychloride, of the same consistency used in filling pulp-canals, was
placed in the funnel portion of the small tube. It soon hardened, but

the coagulating process was marked upon the albumen. It began
immediately and has continued without interruption to the present
time. The line of demarcation between the oxychloride and the
coagulation is distinctly shown. This, probably, is one of the most
satisfactory of the tests, as it abundantly proves that contact with
albumen is all that is necessary to produce coagulation with zinc
chloride, and if this be possible out of the mouth, how much greater
must it be under more favorable conditions in the tooth.
    Caustic potash was experimented upon not as a coagulant, but
to observe the effect on albumen and gelatin. Though several
tests were made, no visible results were produced, though this does
not antagonize the recognized quality of this agent as one of the
most deeply penetrating and uncontrollable caustics used on the
tissues.
    The action of nitrate of silver in repeated tests was rather a
surprise. It has generally been regarded as a superficial coagulant,
but in every instance it has proved deeply penetrating, and coagu-
lating with rapidity and certainty, very nearly equal to zinc chlo-
ride. This fact assumes some importance in connection with the
use of this agent in teeth. Its rapid penetration raises the ques-
tion, Can we use it without danger to the pulp in posterior teeth as
has been recommended? At present I am not prepared to answer
this question, but it seems as though a risk equal to that assumed
in the use of zinc chloride is taken when placed in children’s teeth
for the prevention of caries.
    The experiments were extended to the penetration of the tooth
structure by a number of coagulating agents. A large number of
teeth were kept under the action of these, the pulp-canals being
first slightly enlarged and filled with the agent daily. The result
has not been entirely satisfactory, microscopic examination shows
decided action throughout the dentine, tbe tubes, in several sections
being nearly obliterated and indicated only by fine lines; but while
this demonstrates a positive change in the organic contents of the
tubes, it does not absolutely show that this has been caused by tbe
coagulant. Thus far I have been unable to carry the stain given
the agent along with the coagulation. Silver nitrate in several
sections penetrated in seven days two-thirds the length of the tubes,
but the extreme discoloration made it impossible to follow the indi-
vidual tubes except at the extreme limit of coloration. Tests were
made with a variety of stains, but with no result, the coagulation
invariably separating from the stain. When the color can be car-
ried along with the coagulation, it will visually show what may

be regarded as absolutely true, that the coagulant is carried in the
dentinal tubes as effectually as in those exhibited.
    In the specimens prepared for the microscope, the evidence is
positive to the trained eye that every tube is filled with coagulated
organic matter, and this has been so frequently repeated, and with
precisely the same results, that I have no hesitation in accepting it
as a fact. I failed, however, to observe any change in the cementum,
and I am, therefore, led to doubt the possibility of any coagulating
effect in that tissue by any of the agents used.
    An attempt was made to verify Dr. Kirk’s experiment of cement-
ing a tube in a tooth, sealing the foramen, and then filling the tube
with an active coagulant, as zinc chloride. Six perfectly fresh teeth
were taken, tubes cemented, and foramen closed. The tubes were
of varying length, the fluid in them ranging from a column of 7 to
18 centimetres. These were placed in a second tube filled with egg
albumen. The result in four was that coagulation began after several
hours at points indicating leakage. One of these was removed, the
leak covered with paraffin, and reinserted. This has remained em-
bedded in the albumen for over a month without any result. In
two cases the leak evidently was not through defective manipula-
tion, but appeared to be from an invisible crack in the enamel and
at the bifurcation of molar roots. The two specimens exhibited will,
I think, demonstrate the conclusions arrived at by microscopic tests,
that zinc chloride, the agent used, cannot penetrate through the
cemental tissue. If this cannot be done by the force of the column
of fluid, it certainly cannot by diffusion.
    The error, it seems to me, of Dr. Kirk’s experiment, lies in the
fact that allowance was not made for leakage. It is well known to
all histological workers that teeth may be more or less penetrated
by cracks. These furnish a clear passage for any fluid forced into
the tooth. Fresh teeth are not so liable to this defect, demonstrated
by the specimens exhibited.
    In the paragraph quoted from a portion of my remarks in 1889,
I stated my faith in coagulation as a remedy for discoloration and
an effectual barrier to the ingress of micro-organisms into the den-
tinal tubes. I am still of the same opinion, the only modification
I would make of these views then expressed would be that I fear
the possibility of the action of the zinc chloride upon the perice-
mentum through its penetrating power, passing out through the
foraminae, if more than one exists. My observations, as heretofore
stated, do not show that the use is at all dangerous if care be taken
to close the upper third of the canals thoroughly before placing

the coagulant in the tooth. Clinically, I have observed in one case
only an action upon the pericementum, which I have been led to
attribute to the zinc chloride passing through the foramen. The
large clinical experience in filling root-canals with oxychloride of
zinc, now covering many years and a large number of practitioners,
seems to show that it can be used without risk, provided proper
precautions be taken.
    In the use of zinc chloride as an obtundent of sensitive dentine
there cannot be two opinions. The experiments demonstrate, be-
yond cavil, that this agent is exceedingly dangerous to the life of
the pulp, and should be abandoned for that purpose.
    While it is recognized that the experiments are by no means
exhaustive, I regard them as demonstrating the incorrectness of
the views quoted, and must further regard them as placing the
question on an intelligent basis, and perhaps adding something to
our knowledge as to the relative penetrating power of coagulants.
    I am indebted largely to Mr. F. McS. Thomas for valuable aid
in the manipulative part of the work, wrhich has necessarily been
of a tedious character.
				

## Figures and Tables

**Plate 1. f1:**
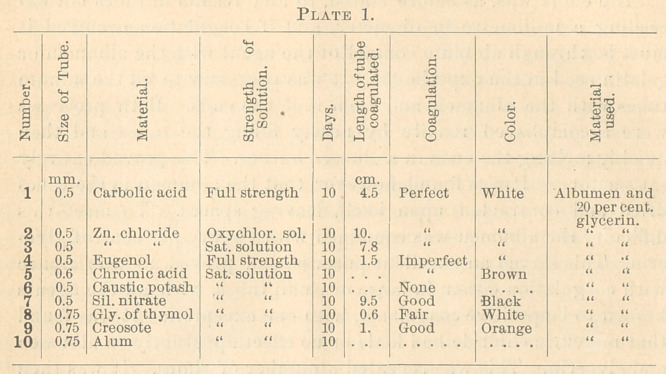


**Plate 2. f2:**
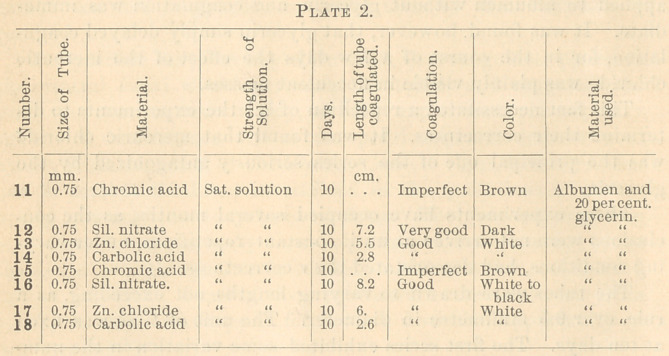


**Figure f3:**
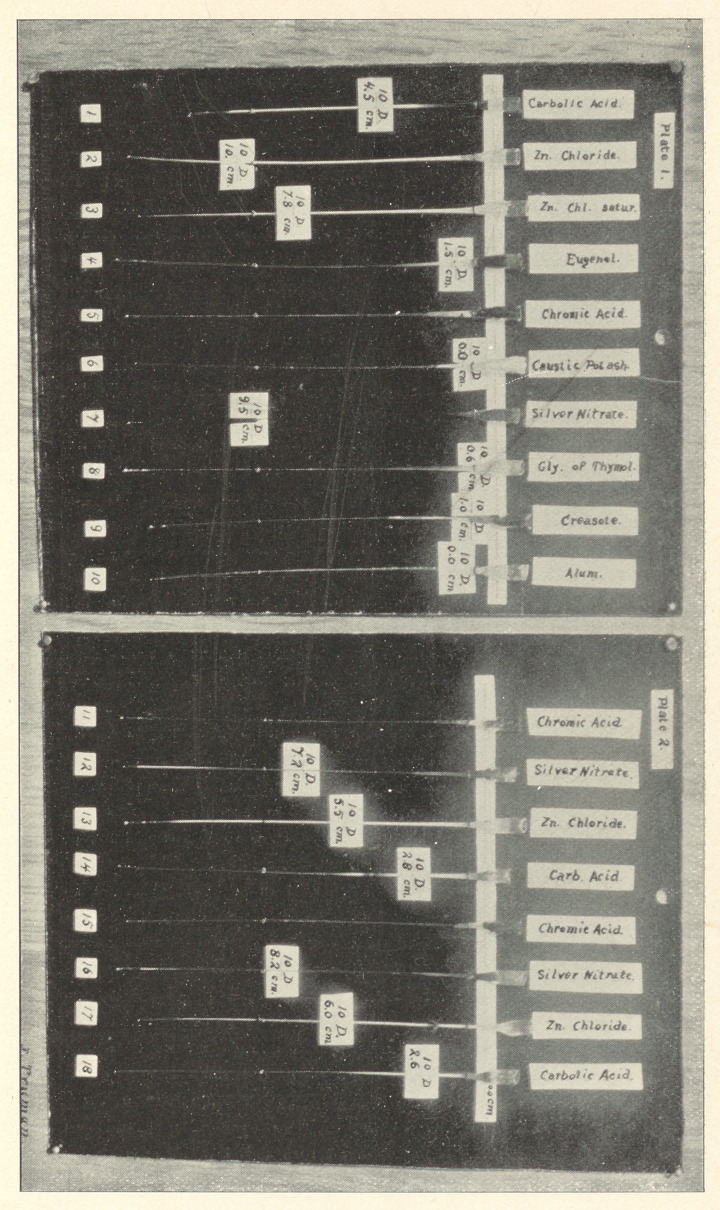


**Figure f4:**